# A Molecular Electron Density Theory Study of the Domino [4+2]/[3+2] Cycloaddition Leading to a Tricyclic 1,2-Oxazine Nitrones

**DOI:** 10.3390/molecules31152725

**Published:** 2026-08-06

**Authors:** Agnieszka Kącka-Zych, Luis R. Domingo

**Affiliations:** 1 Cracow University of Technology, Faculty of Chemical Engineering and Technology, Department of Organic Chemistry and Technology, Warszawska 24, 31-155 Cracow, Poland; 2 Independent Researcher, Av. Tirso de Molina 20, 46015 Valencia, Spain

**Keywords:** bicyclic 1,2-oxazines, [4+2] cycloaddition reaction, [3+2] cycloaddition reaction, domino reaction, molecular electron density theory

## Abstract

The reaction of 2,5-dimethylfuran (DMF) with two equivalents of α-nitrosostyrene (NS) leading to a tricyclic 1,2-oxazine nitrone (TON) has been examined theoretically at the ωB97X-D/6-311G(d,p) computational level. In this case, the domino process should be considered: (i) a [4+2] cycloaddition (42CA) between DMF and NS yielding a bicyclic 1,2-oxazine (BO); and (ii) a second formal [3+2] cycloaddition (32CA) reaction between BO and NS yielding the final TON. Analysis of the reactivity indices shows that DMF and BO generated along the first 42CA reaction are strong nucleophiles, while NS is a strong electrophile. The first 42CA reaction takes place according to a one-step mechanism. In turn, the second 32CA proceeds according to a two-step mechanism through the zwitterionic intermediate **ZW**. It is worth noting that the activation enthalpy of the significant point (**TS-1mn**) of the 42CA is very low, 2.17 kcal·mol^−1^, the reaction being strongly exothermic by −37.71 kcal·mol^−1^. This cycloaddition reaction is completely *meta* regioselective and *endo* stereoselective. The first step of the formal 32CA reaction has an activation enthalpy of 8.48 kcal·mol^−1^ (**TS-21**), the overall domino process being strongly exothermic by −40.34 kcal·mol^−1^. Both **TS-1mn** and **TS-21** are associated with highly asynchronous single bond processes. The high global electron density transfer (GEDT) found at both TSs, higher than 0.32e, points out the high polar character of these cycloaddition reactions, classified as reverse electron density flux (REDF). Electron Localization Function (ELF) analysis of **TS-1mn** and **TS-21** shows that the C2-C3 and C3-N5 bonds are not formed at the same time, while in the intermediate **ZW** we observed the creation of one of them.

## 1. Introduction

**Scheme 1:**
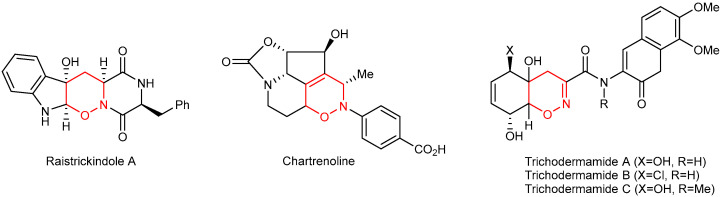
Examples of bioactive 1,2-oxazine derivatives [5,6,7,8,9,10].

Titled 1,2-oxazine derivatives are a very important class of compounds belonging to nitrogen-oxygen heterocycles, which are key in organic chemistry and medicine [1,2,3,4]. The 1,2-oxazine core has been discovered in natural products, making 1,2-oxazine derivatives important compounds (Scheme 1) [5,6,7,8,9,10].

**Scheme 2:**
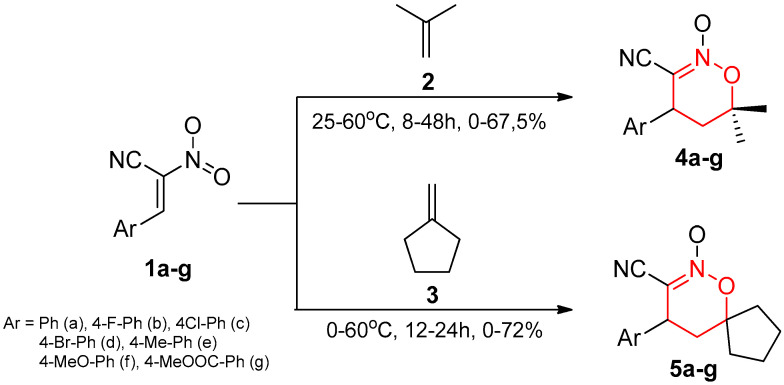
42CA reactions of the (2E)-3-aryl-2-nitroprop-2-enenitriles (**1a**–**g**) and isobutene (**2**) and methylenecyclopentane (**3**) [12,13].

Many reactions leading to 1,2-oxazine moieties arise via [4+2] cycloaddition (42CA) reactions [11]. For example, a number of 1,2-oxazine N-oxide derivatives were obtained in the 42CA reaction between various nitriles and isobutene [12] or methylenecyclopentane [13]. 1,2-Oxazine N-oxide derivatives were obtained in accordance with the principles of green chemistry and selectivity, with good yields reaching 72% (Scheme 2).

**Scheme 3:**
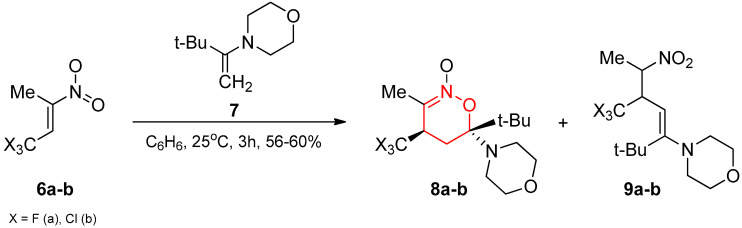
42CA reactions between 1,1,1-trihalo-3-nitrobut-2-ene (**6a**–**b**) and 3,3-dimethyl-2-morpholinobutene (**7**) leading to 1,2-oxazine N-oxides (**8a**–**b**) [14].

1,2-Oxazine N-oxides can also be obtained in 42CA reactions between 1,1,1-trihalo-3-nitrobut-2-ene (**6a**–**b**) and 3,3-dimethyl-2-morpholinobutene (**7**) (Scheme 3) [14]. It was found that the reaction of nitroalkene **6a**–**b** with **7** under kinetic control conditions gives 1,2-oxazine N-oxides (**8a**–**b**) in 56–60% yields. In this reaction, in addition to 1,2-oxazine N-oxides, a noncyclic adduct (**9a**–**b**) is also formed.

**Scheme 4:**
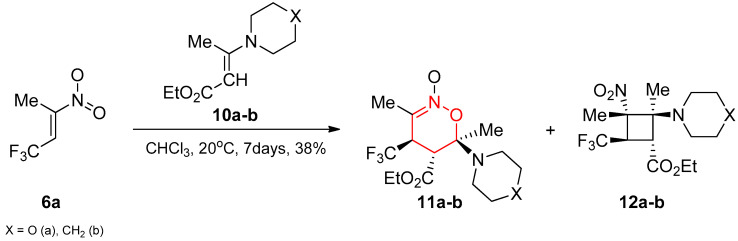
42CA reactions of the 1,1,1-trifluoro-3-nitrobut-2-ene (**6a**) and enamines (**10a**–**b**) yielding 1,2-oxazine N-oxide derivatives (**11a**–**b**) [15].

Another example of a 42CA reaction leading to 1,2-oxazine N-oxides (**11a**–**b**) is the reaction between 1,1,1-trifluoro-3-nitrobut-2-ene (**6a**) and enamines (**10a**–**b**) (Scheme 4). The authors presented an efficient non-catalyzed 42CA reaction yielding 1,2-oxazine N-oxide derivatives (**11a**–**b**) with a yield of 38% [15]. In this reaction, in addition to 1,2-oxazine N-oxides, the formation of [2+2] cycloadducts (**12a**–**b**) was observed.

**Scheme 5:**
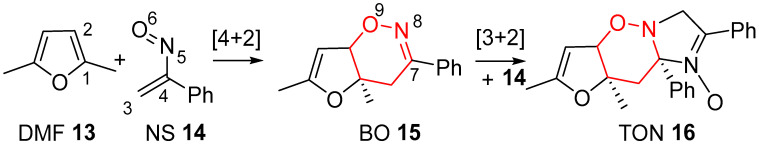
Domino [4+2]/[3+2] cycloaddition reactions between DMF **13** and NS **14** yielding TON **16**.

Mackay et al. [16] studied the 42CA reaction of 2,5-dimethylfuran (DMF, **13**) with α-nitrosostyrene (NS, **14**) in order to obtain a [4+2] bicyclic compound. However, they found the formation of the bicyclic 1,2-oxazine (BO, **15**) together with the tricyclic 1,2-oxazine nitrone (TON) **16** as a minor product (Scheme 5). The optimum conditions were observed in the reaction of one equivalent of DMF **13** with two equivalents of NS **14**, yielding TON **16** in 50% yield. Creation of the final product TON **16** runs through the domino process consisting of a 42CA reaction of DMF **13** and NS **14** yielding BO **15** and [3+2] cycloaddition (32CA) reaction of the BO **15** and NS **14** yielding the final TON **16**. It was proven that reversal of TON **16** to BO **15** did not occur under the process conditions [16].

Herein, the domino reaction of DMF **13** with an excess of NS **14** yielding the tricyclic TON **16**, experimentally studied by Mackay et al. [16], is investigated within the Molecular Electron Density Theory [17] (MEDT) in order to explain the experimental outcomes (see Scheme 5). This MEDT study is structured into four distinct sections: (i) first, an Electron Localization Functions [18] (ELF) analysis of the electronic structure of reagents participating in this domino process is conducted; (ii) next, analysis of the DFT-based reactivity indices [19,20] of the reagents is performed in order to predict their reactivity in polar processes; (iii) in this section, the potential energy surface (PES) associated with the domino [4+2]/[3+2] cycloaddition reaction between DMF **13** and NS **14** yielding TON **16** is explored; and finally, (iv) an ELF topological analysis of the transition state structures (TSs) and intermediate involved in this domino reaction is performed.

## 2. Results and Discussion

### 2.1. Topological Analysis of the ELF and NPA of DMF ***13***, NS ***14*** and BO ***15***

**Figure 1:**
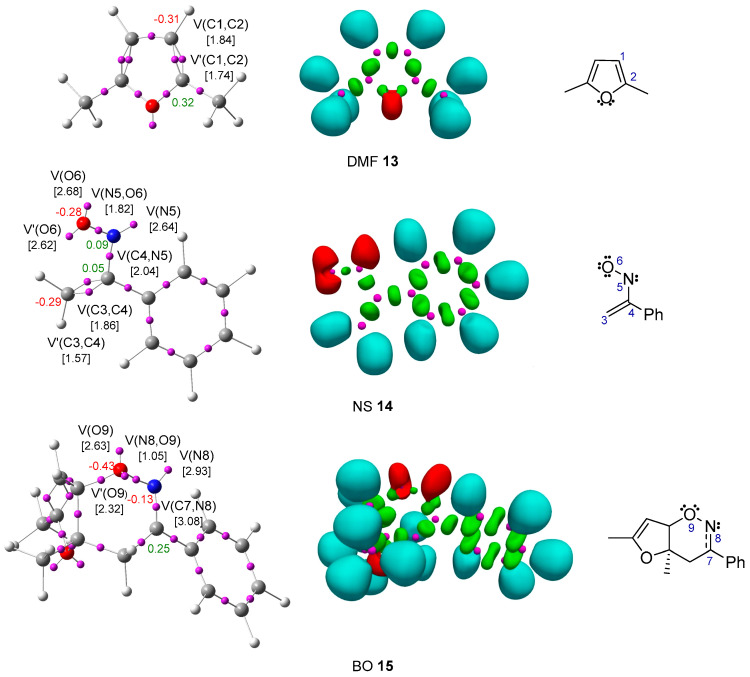
ELF picture together with the proposed Lewis-like structure of reagents: DMF **13**, NS **14,** and BO **15**. ELF valence basin population and natural atomic charges are given in average number of electrons, e.

In order to get to know the electronic structure of the reagents DMF **13**, NS **14,** and BO **15,** we conducted the topological analysis of the ELF [18], which allows the determination of the electron density distribution [21,22], providing a connection between electronic structure and reactivity. The results of the ELF analysis are pictured in Figure 1. Full characterization of the compounds participating in the domino [4+2]/[3+2] cycloaddition leading to a tricyclic 1,2-oxazine nitrone is available in the Supplementary Materials in Table S2 on page S5.

ELF analysis of the first reagent, DMF **13,** points out the occurrence of two disynaptic basins, V(C1,C2) and V’(C1,C2), integrating 1.84 e and 1.74 e, respectively, which are attributable to the furan C1-C2 double bond (Figure 1). It is worth noting that DMF **13** is a symmetric molecule that has in its structure two V(C1′,C2′) and V’(C1′,C2′) disynaptic basins connected with the C1′-C2′ double bond.

ELF of NS **14** displays the presence of two disynaptic basins, V(C3,C4) and V’(C3,C4), integrating 1.86 e and 1.57 e, respectively, characterizing the C3-C4 double bond, and two disynaptic basins, V(C4,N5) and V(N5,O6), integrating 2.04 e and 1.82 e, respectively. These disynaptic basins are relevant to the C4-N5 and N5-O6 single bonds. The N5 nitrogen presents a V(N5) monosynaptic basin with a population of 2.64 e, which refers to the non-bonding electron density at N5. Interestingly, the C3–C4–N5 framework of NS **14** does not possess the structure of the three-atom-component (TAC) participating in 32CA reactions, in which the central atom is a nitrogen, oxygen, sulfur, or phosphorus heteroatom.

ELF topological analysis of BO **15** exhibits the appearance of two disynaptic basins, V(C7,N8) and V(N8,O9), integrating 3.08 e and 1.05 e, respectively, connected with a low-populated C7-N8 double bond and a low-populated N8-O9 single bond. (Figure 1). ELF of BO **15** also indicates the presence of three V(N8), V(O9), and V’(O9) monosynaptic basins related to the non-bonding electron density located on the N8 and O9 atoms.

We also decided to get to know the electron density distribution in the DMF **13**, NS **14** and BO **15** structures using NPA [23,24] analysis. NPA of DMF **13** suggests that the C1 carbon is positively charged by 0.32 e, while the C2 carbon is negatively charged by −0.31 e. The NPA analysis of NS **14** shows that the C3 carbon and O6 oxygen are negatively charged by −0.29 e and −0.28 e, respectively, while the C4 and N5 atoms practically present a null charge. Finally, NPA of BO **15** suggests that while the N8 and O9 atoms are negatively charged by −0.13 and −0.43 e, the C7 carbon is positively charged by 0.25 e.

### 2.2. Analysis of the DFT-Based Reactivity Indices at the GS of the Reagents

In the next step, we performed a DFT-based reactivity indices [19,20] study to determine the reactivity of the reactants in the reaction leading to TON **16**. The reactivity indices were computed at the B3LYP/6-31G(d) computational level, as it was used to establish electrophilicity and nucleophilicity scales [20]. Since the DFT-based reactivity indices depend on the chosen level of theory, in particular on the functional, and were originally defined and calibrated at the B3LYP/6-31G(d) level of theory, this computational level was selected to ensure direct comparison with established empirical reactivity scales [20]. The values of the reactivity indices of the reagents, DMF **13**, NS **14** and BO **15,** are exhibited in Table 1.

As shown in Table 1, the electronic chemical potential [25] µ of DMF **13**, −2.39 eV, and BO **15**, −3.46 eV, are higher than that of NS **14**, −4.50 eV. According to this, along polar cycloaddition reactions, the global electron density transfer [26] (GEDT) will take place from DMF **13** and BO **15** toward NS **14** in reactions classified as reverse electron density flux (REDF) [27].

DMF **13** has an electrophilicity ω index [28] of 0.46 eV, and a nucleophilicity *N* index [29] of 3.63 eV, being categorized as a marginal electrophile and as a strong nucleophile within the standard electrophilicity and nucleophilicity scales [20]. NS **14**, based on its electrophilicity ω index, 3.12 eV, and its nucleophilicity *N* index, 3.00 eV, is categorized as a strong electrophile and strong nucleophile. Finally, BO **15** with a ω = 1.23 eV and *N* = 3.22 eV is categorized as a moderate electrophile and as a strong nucleophile. Consequently, while NS **14** will participate in cycloaddition reactions as a strong electrophile, DMF **13** and BO **15** will participate as strong nucleophiles in highly polar reactions.

**Figure 2:**
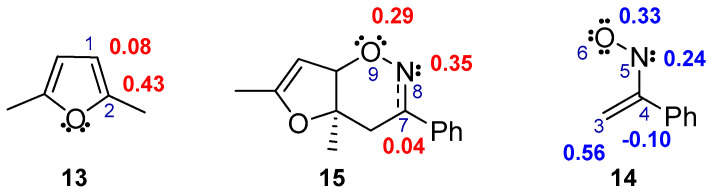
Representation of nucleophilic $P_{k}^{-}$ (for DMF **13** and BO **15**) and electrophilic $P_{k}^{+}$ Parr functions (for NS **14**).

Next, we decided to carry out the study of the nucleophilic $P_{k}^{-}$ and electrophilic $P_{k}^{+}$ Parr functions [30] which characterize the local reactivity in polar reactions, allowing us to explain the regioselectivity in polar cycloaddition reactions. The results of the study for reagents DMF **13**, NS **14** and BO **15** are given in Figure 2.

Analysis of the nucleophilic $P_{k}^{-}$ Parr functions at DMF **13** points that the C2 carbon, $P_{k}^{-}$ = 0.42, is the most nucleophilic center in this structure (see Figure 2). On the other hand, the N8 nitrogen, $P_{k}^{-}$ = 0.35, and O9 oxygen, $P_{k}^{-}$ = 0.29, are the highly nucleophilically activated centers of BO **15**. Finally, analysis of the electrophilic $P_{k}^{+}$ Parr functions at NS **14** states that the C3 carbon $P_{k}^{+}$ = 0.56, is the most electrophilic center in this structure.

Therefore, the most favorable electrophile–nucleophile two-center interaction along the 42CA reaction between DMF **13** and NS **14** will take place between the most nucleophilic center of the nucleophilic DMF **13**, the C2 carbon, and the most electrophilic center of the electrophilic NS **14**, the C3 carbon, in clear agreement with the total *meta* regioselectivity experimentally observed when only one equivalent of the two reagents was used [16]. Analysis of the subsequent formal 32CA reaction between BO **15** and NS **14** suggests that the initial two-center interaction will take place between the nucleophilic N8 nitrogen of BO **15** and the most electrophilic center of the electrophilic NS **14**, the C3 carbon. Note that although the O9 oxygen of BO **15** is more nucleophilically activated than the N8 nitrogen, it appears the initial C3-O9 two-center interaction cannot evolve to a final adduct.

It is worth noting that the C3 carbon of NS **14**, which is the most electrophilic center of this species, $P_{k}^{+}$ = 0.56, shows a high negative charge similar to that of the O6 oxygen, −0.29 e (see Figure 1). This behavior confirms the thesis proposed by Domingo in 2013, which indicates that the local electrophilic/nucleophilic behaviors of organic molecules are not simply caused by charges [31].

### 2.3. The Potential Energy Surfaces (PESs) Study of the Domino [4+2]/[3+2] Cycloaddition Reaction Between DMF ***13*** and NS ***14*** Yielding TON ***16***

**Scheme 6:**
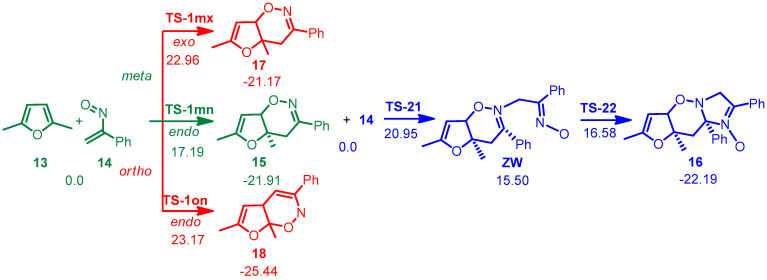
Domino [4+2]/[3+2] cycloaddition reaction between DMF **13** and NS **14** yielding TON **16**. The 42CA reaction path has been highlighted in green, while the 32CA reaction path has been highlighted in blue. The ωB97X-D/6-311G(d,p) Gibbs free energies in benzene, with respect to **13** + **14** or **15** + **14**, are given in kcal·mol*^−^*^1^.

The reaction between DMF **13** and NS **14** yielding TON **16** is a domino process that comprises two consecutive cycloaddition reactions (see Scheme 6): (i) a 42CA reaction between DMF **13** and NS **14** yielding BO **15**; and (ii) a formal 32CA reaction between BO **15** and NS **14** yielding the final product TON **16**. Due to the nonsymmetry of both reagents, two pairs of regioisomeric reaction channels, the *ortho* and *meta*, and two pairs of stereoisomeric reaction paths, the *endo* and *exo*, are feasible in the first 42CA reaction. Due to the high regioselectivity found in polar 42CA reactions [32,33], only the *endo* reaction path along the more unfavorable *ortho* reaction path was studied. Analysis of the stationary points involved in the titled reactions shows that the first 42CA occurs according to the one-step mechanism. In turn, the second 32CA is accomplished by a two-step mechanism with the production of a zwitterionic intermediate ZW (see Scheme 6). The relative enthalpies are given in Scheme 6, while the thermodynamics are given in Table S1 in the Supplementary Materials.

Some compelling conclusions can be obtained from the relative enthalpies given in Scheme 6: (i) the first 42CA reaction between DMF **13** and NS **14** presents a Gibbs free energy of 17.19 kcal·mol^−1^; (ii) this cycloaddition reaction is completely *endo* selective to be located the *exo* **TS-1mx** 5.77 kcal·mol^−1^ above the *endo* **TS-1mn**; (iii) the 42CA reaction is completely regioselective to be located the *ortho/endo* **TS-1on** 5.98 kcal·mol^−1^ above the *meta/endo* **TS-1mn**; (iv) the first 42CA reaction is highly exothermic by −21.91 kcal·mol^−1^. Consequently, the first 42CA reaction is irreversible, and kinetically controlled; (v) from BO **15** plus NS **14**, the first step of the second formal 32CA reaction has a Gibbs free energy of 20.95 kcal·mol^−1^, formation of the intermediate **ZW** being slightly endothermic by 15.50 kcal·mol^−1^; (vi) from this intermediate, the second step of the 32CA reaction has an unappreciable Gibbs free energy of 1.08 kcal·mol^−1^; and finally, (vii) this 32CA reaction is also exothermic by −22.19 kcal·mol^−1^. Consequently, the second formal 32CA reaction is also irreversible.

**Figure 3:**
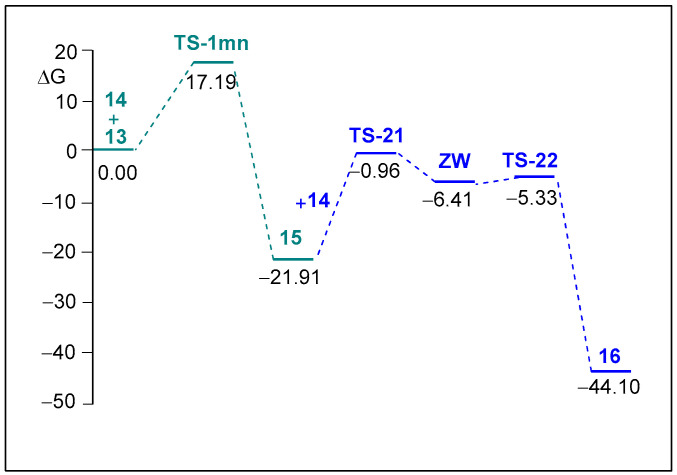
ωB97X-D/6-311G(d,p) Gibbs free energy profile, in kcal·mol^−1^, for the domino reaction between DMF **13** and NS **14**. The 42CA reaction path has been highlighted in green, while the formal 32CA reaction path has been highlighted in blue. Relative Gibbs free energies are given with respect to DMF **13** and NS **14**.

A representation of the Gibbs free energy profiles associated with the domino reaction between DMF **13** and NS **14** yielding TON **16** is shown in Figure 3. The activation Gibbs free energy associated with the first 42CA reactions of this domino process is 17.19 kcal·mol^−1^, the reaction being strongly exergonic by −21.91 kcal·mol^−1^, and consequently irreversible. From BO **15** plus NS **14**, the activation Gibbs free energy associated with the nucleophilic attack of BO **15** on NS **14** is 20.95 kcal·mol^−1^; intermediate **ZW** is found 5.45 kcal·mol^−1^ below **TS-21**. The activation Gibbs free energy associated with the second step of the stepwise 32CA reaction is very low, 1.08 kcal·mol^−1^, in agreement with the earlier character of **TS-22**. Consequently, the first step of the formal 32CA reaction is the rate-determining step of this domino process. Finally, the overall domino reaction yielding TON **16** is strongly exergonic by −44.1 kcal·mol^−1^.

The activation Gibbs free energy associated with the second 32CA reaction via **TS-21** is only 3.76 kcal·mol^−1^ higher in Gibbs free energy than that associated with the 42CA reaction of DMF **13** and NS **14** via **TS-1mn**. This behavior is in agreement with the fact that in the earlier experiments in which one equivalent of the two reagents was used, the TON **16** was obtained in low yield [16]. However, due to the strong exergonic character of the formation of BO **15**, in the reaction conditions in which two equivalents of NS **14** were used, the domino reaction progresses via **TS-21** to yield the final TON **16**. Finally, the strong exergonic character of the formation of TON **16** from BO **15** plus NS **14**, −22.19 kcal·mol^−1^, makes the formal 32CA reaction also irreversible in agreement with the experimental outcomes [16].

**Figure 4:**
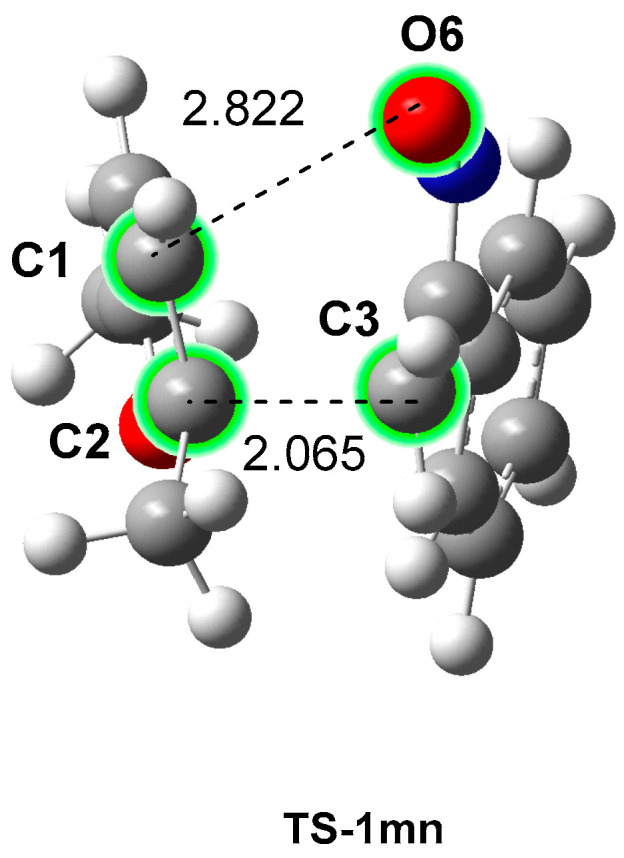
ωB97X-D/6-311G(d,p) optimized geometry of **TS-1mn** involved in the 42CA between DMF **13** and NS **14**.

**Figure 5:**
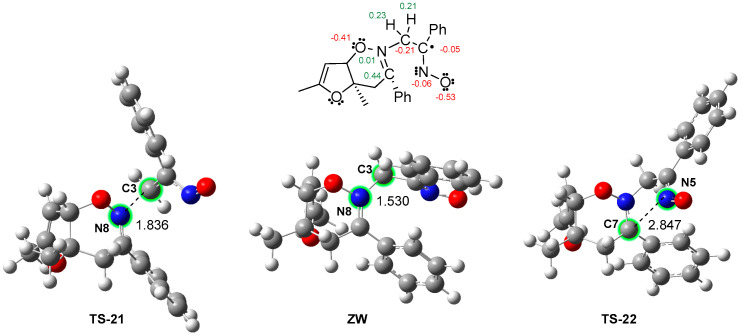
ωB97X-D/6-311G(d,p) (PCM) optimized geometries of **TS-21**, **ZW** and **TS-22** involved in the formal 32CA between BO **15** and NS **14**. NPA analysis for the ZW structure.

The geometries of the **TSs** and intermediate **ZW** involved in the domino reaction between DMF **13** and NS **14** are shown in Figure 4 and Figure 5. At **TS-1mn,** involved in the 42CA reaction between DMF **13** and NS **14,** the distances between the two pairs of interacting atoms are 2.065 Å (C2-C3) and 2.822 Å (C1-O6). These distances point out a highly asynchronous single bond formation, which is associated with the nucleophilic attack of the C2 carbon of DMF **13** at the most electrophilic center of NS **14**. The distance between the C2 and C3 carbons, which is higher than 2.0 Å, indicates that the C2-C3 single bond formation has not begun at this TS [26] (as discussed later).

At **TS-21,** associated with the nucleophilic attack of the N8 nitrogen of BO **15** on the C3 conjugated position of the strong electrophilic NS **14,** the C3-N8 distance is 1.836 Å (see Figure 5). At the intermediate **ZW,** the C3-N8 distance has been reduced to 1.530 Å, indicating the formation of the first C3-N8 single bond. Finally, the C7-N5 distance at **TS-22**, 2.847 Å, points out the earlier nature of this TS.

Interestingly, electrophilic NS **14** acts as the heterodiene in the first 42CA reaction, and as a formal TAC in the second 32CA reaction. In both cycloadditions, the reaction begins with the nucleophilic attack of the C2 carbon of DMF **13** or the N8 nitrogen of BO **15** on the C3 conjugated position of the strong electrophilic NS **14** via high asynchronous TSs. Formation of the [4+2] or [3+2] cycloadducts takes place during the ring-closure step, which is associated with the formation of the second single bond at the final stage of the reactions via a non-concerted one-step two-stage mechanism or a two-step mechanism.

Finally, the GEDT [26] at the **TS-1mn** and **TS-21** was evaluated to characterize the polar character of the titled reactions leading to tricyclic 1,2-oxazine nitrones. The GEDT values computed at the TSs, 0.32 e at **TS-1mn** and 0.34 e at **TS-21**, point to the high polar character of these cycloaddition reactions as a consequence of the strong electrophilic character of NS **14** and the strong nucleophilic character of DMF **13** and BO **15** (see Table 1). The high GEDT taking place at **TS-1mn** accounts for the very low activation enthalpy associated with this polar hetero Diels–Alder reaction.

The electron density, which fluxes from DMF **13** and BO **15**, acting as the ethylene components, towards NS **14**, acting as the heterodiene in the 42CA reaction or the formal TAC in the 32CA reaction, classifies these polar cycloaddition reactions as REDF [27] in complete agreement with the previous analysis of the DFT-based reactivity indices. The NPA indicates that the positive charge is located at the C4 carbon and hydrogens connected with the C3 carbon (Figure 5). The N5 nitrogen shows a negligible positive charge of 0.01 e. On the other hand, the negative charges are located on atoms C3, C4, N5, and O6, which come from another molecule **14**, attached in the 32CA. The very high GEDT and NPA computed at the intermediate **ZW**, 0.57 e, point out the high zwitterionic character of this species.

### 2.4. ELF Topological Analysis of the TSs and Intermediate ZW Involved in the Domino [4+2]/[3+2] Cycloaddition Reactions Between DMF ***13*** and NS ***14***

Finally, the electronic structures of the TSs and intermediate ZW associated with the domino [4+2]/[3+2] cycloaddition reaction between DMF **13** and NS **14** were investigated by performing an ELF topological analysis.

#### 2.4.1. ELF Topological Analysis of the **TS-1mn** Involved in the 42CA Reaction of DMF **13** with NS **14**

**Figure 6:**
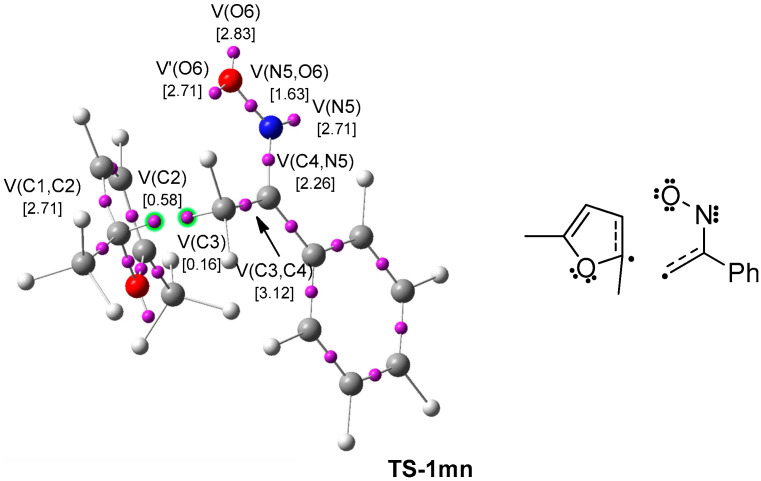
ELF basin attractor positions, along with the most relevant valence basin populations of **TS-1mn** and the proposed Lewis-like structure associated with the 42CA reaction between DMF **13** and NS **14**. The electron populations are the average number of electrons, e.

At the beginning of the analysis, we decided to analyze the electron structure of the **TS-1mn** that takes part in the 42CA reaction between DMF **13** and NS **14**. The results of the analysis are presented in Figure 6.

ELF of **TS-1mn** shows the presence of four disynaptic basins, V(C1,C2), V(C3,C4), V(C4,N5), and V(N5,O6), with populations of 2.71, 3.12, 2.26, and 1.63 e, respectively. The two V(C1,C2) and V(C3,C4) disynaptic basins are connected with two depopulated C1-C2 and C3-C4 double bonds, while the V(C4,N5) and V(N5,O6) disynaptic basins are related to a populated C4-N5 and a depopulated N5-O6 single bond (Figure 6). The most relevant feature of the ELF of **TS-1mn** is the presence of two V(C2) and V(C3) monosynaptic basins integrating 0.58 and 0.16 e, respectively. Note that these monosynaptic basins are formed in the most nucleophilic center of DMF **13**, the C2 carbon, and the most electrophilic center of NS **14**, the C3 carbon, in agreement with the previous analysis of the Parr functions [30]. These monosynaptic basins are related to the creation of the *pseudoradical* centers [34] at the C2 and C3 carbons, which are required for the subsequent C2-C3 single bond formation in a later stage of the reaction [26]. ELF of **TS-1mn** also shows the presence of two V(O6) and V’(O6) monosynaptic basins connected with the non-bonding electron density located on the O6 oxygen. The absence of any V(C2,C3) disynaptic basin indicates that the formation of the new C2-C3 single bond has not begun at **TS-1mn**.

#### 2.4.2. ELF Topological Analysis of the **TSs** and Intermediate **ZW** Involved in the Formal 32CA Reaction of BO **15** with NS **14**

**Figure 7:**
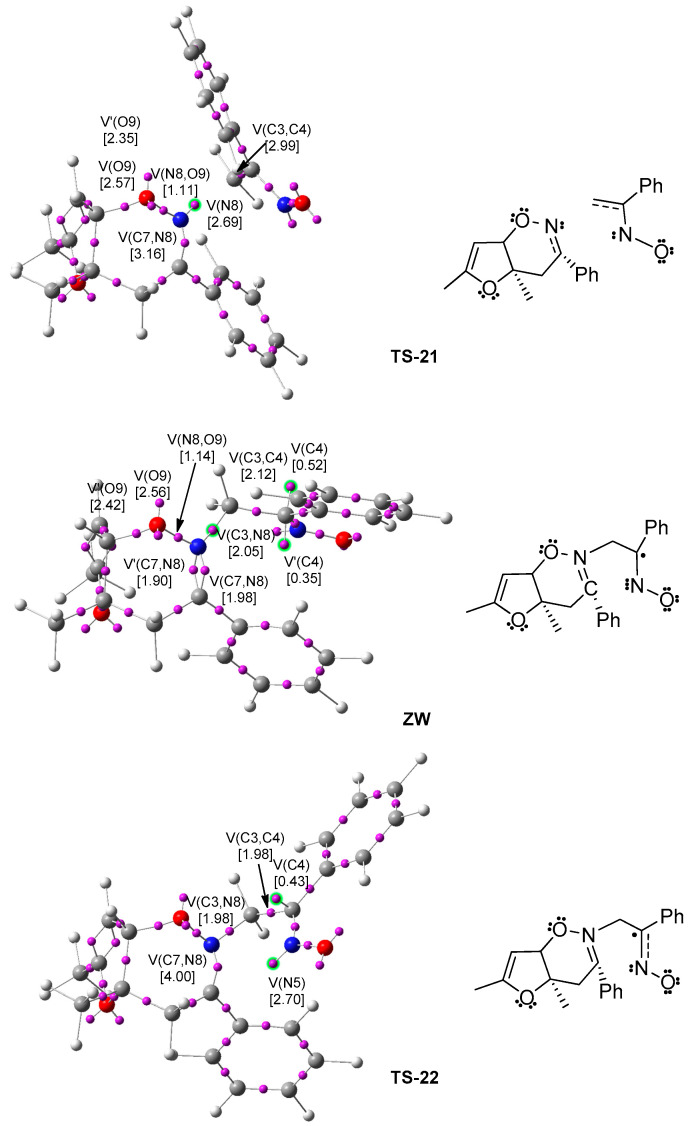
The most important ELF basin attractor for **TS-21**, intermediate **ZW,** and **TS-22** connected with 32CA leading to TON **16**. The electron populations are the average number of electrons, e.

Next, the electronic structures of **TS-21**, intermediate **ZW,** and **TS-22** connected with the 32CA reaction of BO **15** and NS **14** were investigated. The results of the analysis are presented in Figure 7.

The ELF of **TS-21** reveals the presence of two disynaptic basins, V(C7,N8) and V(C3,C4), integrating 3.16 and 2.99 e, respectively, which are associated with the depopulated C7-N8 and depopulated C3-C4 double bonds, and one V(N8,O9) disynaptic basin, integrating 1.11 e, associated with a depopulated N8-O9 single bond. ELF also shows the presence of three V(N8), V(O9), and V’(O9) monosynaptic basins related to non-bonding electron density present on the N8 nitrogen and O9 oxygen. The V(N8) monosynaptic basin is required for the subsequent formation of a single bond between N8 and C3 centers. In spite of the short C3-N8 distance (see Figure 5), the absence of any V(C3-N8) disynaptic basin indicates that the formation of the new C3-N8 single bond has not begun at this TS.

ELF of the intermediate **ZW** shows the presence of a new V(C3,N8) disynaptic basin integrating 2.05 e (Figure 7). This valence basin is associated with the formation of the first C3-N8 single bond along this stepwise formal 32CA reaction. ELF of **ZW** also shows the presence of two new V(C4) and V’(C4) monosynaptic basins at the NS framework integrating 0.52 e and 0.35 e, respectively. These monosynaptic basins indicate the presence of one C4 *pseudoradical* carbon at **ZW**. Note that this *pseudoradical* C4 center will be involved in the formation of the C-N double bond present at the final nitrone TON **16**.

Finally, ELF of **TS-22** is very similar to that of **ZW**. ELF exhibits the appearance of two V(C4) and V’(C4) monosynaptic basins with an initial population of 0.87 e. No V(C3) monosynaptic basin, which is required for the subsequent C3-N8 single bond creation, is observed at this TS. This behavior asserts the early nature of **TS-22**.

## 3. Calculation Details

In the presented MEDT studies, the ωB97X-D [35] functional was used together with the 6-311G(d,p) basis set [36]. All electronic structure calculations were carried out using the Gaussian 16 suite of programs [37]. The PL-Grid infrastructure (“Ares” supercomputer) at the national computer center “Cyfronet” was used. All studies were performed according to a well-known protocol previously used [38,39,40,41,42,43]. Detailed information is presented in the Supplementary Materials on page S2.

## 4. Conclusions

The reaction between DMF **13** and NS **14** yielding TON **16**, experimentally reported by Mackay et al. [16], has been studied within MEDT at the ωB97X-D/6-311G(d,p) computational level in benzene. This reaction is a domino process that comprises two consecutive cycloadditions: (i) a 42CA between DMF **13** and NS **14** yielding BO **15**; and (ii) a second formal 32CA reaction between BO and NS **14** yielding the final TON **16**.

Analysis of the DFT-based reactivity indices of the reagents DMF **13**, NS **14,** and BO **15** confirms that DMF **13** and BO **15** are strong nucleophiles, while NS **14** is a strong electrophile. Analysis of GEDT of the titled reactions indicates their polar nature and classification as REDF. The study of the Parr functions permits pointing out the most nucleophilic center of DMF **13** and the most electrophilic center of NS **14**, allowing for the explanation of the regioselectivity and the asynchronicity of the first 42CA reaction.

Scrutiny of the PES of this domino reaction permits that the first 42CA reaction between DMF **13** and NS **14** takes place via a one-step mechanism, while the second formal 32CA reaction takes place according to a two-step mechanism with the creation of a zwitterionic intermediate **ZW**. The activation enthalpy related to the 42CA reaction via **TS-1mn** is very low, 2.17 kcal·mol^−1^, the reaction being strongly exothermic by −37.71 kcal·mol^−1^. This 42CA reaction is completely *meta* regioselective and *endo* stereoselective. The first step of the 32CA reaction between BO **15** and NS **14**, which corresponds with the rate-determining step of this domino process, has an activation enthalpy of 8.48 kcal·mol^−1^ (**TS-21**), the overall domino process being strongly exothermic by −40.34 kcal·mol^−1^. Analysis of the Gibbs energy profile of this domino reaction is in complete agreement with the experimental outcomes.

Finally, ELF analysis of **TS-1mn** and **TS-21** shows that the creation of the new C-C and C-N single bonds has not begun at any of the two TSs, while ELF on intermediate **ZW** shows that the new C-N single bond has been formed.

## Figures and Tables

**Table 1:** B3LYP/6-31G(d) reactivity indices, in eV, for DMF **13**, NS **14** and BO **15**.

	μ	η	ω	*N*
NS **14**	−4.50	3.24	3.12	3.00
BO **15**	−3.46	4.88	1.23	3.22
DMF **13**	−2.39	6.21	0.46	3.63

## Data Availability

The original contributions presented in this study are included in the article. Further inquiries can be directed to the corresponding author.

## Supplementary Materials

The following supporting information can be downloaded at: https://www.mdpi.com/article/10.3390/molecules31152725/s1, 1. Quantum-chemical calculations; Table S1: ωB97X-D/6-311G(d,p) enthalpies, H in a.u., entropies S in cal·mol^−1^·K^−1^ and Gibbs free energies, G in a.u., computed in benzene at 25 °C, of the stationary points associated with the domino [4+2]/[3+2] cycloaddition reactions between DMF **13** and NS **14**; Table S2. ωB97X-D/6-311G(d,p) computed total energies in benzene, single imaginary frequency of the TSs, and Cartesian coordinates of the stationary points involved in the domino [4+2]/[3+2] cycloaddition reactions between DMF **13** and NS **14**. References [18,19,20,23,24,26,30,35,36,37,44,45,46,47,48] are cited in the supplementary materials.
