# Supplementary material for: A Molecular Electron Density Theory Study of the Domino [4+2]/[3+2] Cycloaddition Leading to a Tricyclic 1,2-Oxazine Nitrones
Source: Molecules. 2026 Aug 6;31(15):2725. doi: 10.3390/molecules31152725 (PMC13468678; doi:10.3390/molecules31152725)
Supplement: Supplementary file 1 [file molecules-31-02725-s001.zip › molecules-4451759-supplementary.pdf]

Supplementary Material

A Molecular Electron Density Theory Study of the Domino [4+2]/[3+2] Cycloaddition Leading to a Tricyclic 1,2-Oxazine Nitrones

Agnieszka Kącka-Zych [1,*] and Luis R. Domingo [2,*]

[1] Cracow University of Technology, Faculty of Chemical Engineering and Technology, Department of Organic Chemistry and Technology, Warszawska 24, 31-155 Cracow, Poland
[2] Independent Researcher, Av. Tirso de Molina 20, 46015 Valencia, Spain
* Correspondence: agnieszka.kacka-zych@pk.edu.pl (A.K.-Z.); luisrdomingo@gmail.com (L.R.D.)

Index

S2 **1. Quantum-chemical calculations.**

S4 **Table S1.** ωB97X-D/6-311G(d,p) enthalpies, H in a.u., entropies S in cal·mol^{-1}·K^{-1} and Gibbs free energies, G in a.u., computed in benzene at 25 ºC, of the stationary points associated with the domino [4+2]/[3+2] cycloaddition reactions between DMF **13** and NS **14**.

S5 **Table S2.** ωB97X-D/6-311G(d,p) computed total energies in benzene, single imaginary frequency of the TSs, and Cartesian coordinates of the stationary points involved in the domino [4+2]/[3+2] cycloaddition reactions between DMF **13** and NS **14**.

.

1. Quantum-chemical calculations

The ωB97X-D [1] functional together with the standard 6-311G(d,p) basis set [2], which incorporates d-type polarization for second-row elements and p-type polarization functions for hydrogens, was employed throughout this Molecular Electron Density Theory (MEDT) study. All electronic structures calculations were carried out using the Gaussian 16 suite of programs [3]. The PL-Grid infrastructure ("Ares" super-computer) at the national computing centre "Cyfronet" was used. All optimized critical points were verified on the basis of the full vibrational analysis. We found that addent molecules, intermediate and products had positive Hessian matrices, while all optimized TSs exhibited only one negative eigenvalue in their Hessian matrices. Intrinsic reaction coordinate (IRC) calculations were performed for all optimized TSs [4]. The obtained IRC trajectories confirmed, without doubt, the postulated nature of the TSs and their role within the energy profile. Solvent effects were studied by modeling the solvent benzene using the polarizable continuum model [5,6] (PCM) with the self-consistent reaction field method, and the stationary points were optimized at ωB97X-D/6-311G(d,p) level of theory. The thermodynamic parameters, i.e., the enthalpies, entropies, and Gibbs free energies, were calculated at 298.15 K and 1 atm in benzene using the standard statistical thermodynamics [2].

The global electron density transfer [7] (GEDT) at the TSs was estimated using the formula: GEDT = Σq_A, where q_A are the atomic charges obtained by a natural population analysis (NPA) [8,9], and the sum is taken over all the atoms of NS framework. The DFT-based reactivity indices [10,11] at the ground state (GS) of the reagents were estimated using equations give in reference 11. The Electron Localization Function (ELF) [12] studies were performed with the TopMod package [13] considering the standard cubical grid of step size of 0.1 Bohr. The ELF molecular geometries and basin attractor positions were visualized using the GaussView program [14].

Computing the nucleophilic P_k^- and electrophilic P_k^+ Parr functions [15] of a molecule was carried out using Gaussian 16 [16]. Once the chemical structure of our target molecule has been defined, the only thing left to do is to optimize its geometry at the desired computational level (i.e. RB3LYP/6-31G(d)/charge 0/multiplicity 1). With the optimized neutral molecular structure at hand, one has to compute the atomic spin density of the radical anion and of the radical cation of the neutral molecule geometry at the same computational level (i.e. single point calculation at UB3LYP/6-31G(d)/charge -1/multiplicity 2 for radical anion and UB3LYP/6-31G (d)/charge +1/multiplicity 2; note that open-shell formalism must be used since they are doublet radicals). In the output of both open-shell calculations, one has to find the Mülliken atomic charges or Natural ones if a NBO calculation has been performed (their results tend to be very similar between them) and collect them separately. Then, we are ready to compute the electrophilic Parr functions from the radical anion atomic charges and the nucleophilic Parr functions from the radical cation ones.

References:

1. Chai, J.-D.; Head-Gordon, M. Long-range corrected hybrid density functionals with damped atom–atom dispersion corrections, *Phys. Chem. Chem. Phys.* **2008**, *10*, 6615-6620.

2. Hehre, W.J.; Radom, L.; Schleyer, P.V.; Pople, J.A. in *Ab initio Molecular Orbital Theory*, Wiley, New York, **1986**.
3. Frisch, M. J.; Trucks, G. W.; Schlegel, H. B.; Scuseria, G. E.; Robb, M. A.; Cheeseman, J. R.; Montgomery, J. A.; Vreven, T. J.; Kudin, K. N.; Burant, J. C.; Millam, J. M.; Iyengar, S.

S.; Tomasi, J.; Barone, V.; Mennucci, B.; Cossi, M.; Scalmani, G.; Rega, N.; Petersson, G. A.; Nakatsuji, H.; Hada, M.; Ehara, M.; Toyota, K.; Fukuda, R.; Hasegawa, J.; Ishida, M.; Nakajima, Y.; Honda, O.; Kitao, O.; Nakai, H.; Klene, M.; Li, X.; Knox, J. E.; Hratchian, H. P.; Cross, J. B.; Adamo, C.; Jaramillo, J.; Gom-perts, R.; Stratmann, R. E.; Yazyev, O.; Austin, A. J.; Cammi, R.; Pomelli, C.; Ochterski, J. W.; Ayala, P. Y.; Morokuma, K.; Voth, G. A.; Salvador, P.; Dannenberg, J. J.; Zakrzewski, V. G.; Dapprich, S.; Daniels, A. D.; Strain, M. C.; Farkas, M. C.; Malick, D. K.; Rabuck, A. D.; Raghavachari, K.; Foresman, J. B.; Ortiz, J. V.; Cui, Q.; Baboul, A. G.; Clifford, S.; Cioslowski, J.; Stefanov, B. B.; Liu, G.; Liashenko, A.; Piskorz, P.; Komaromi, I.; Martin, R. L.; Fox, D. J.; Keith, T.; Al-Laham, M. A.; Peng, C. Y.; Nanayakkara, A.; Challacombe, M.; Gill, P. M. W.; Johnson, B.; Chen, W.; Wong, M. W.; Gonzalez, C.; Pople, J. A. *Gaussian 16* rev A.1 Gaussian Inc, Wallingford CT, **2016**.

4. Fukui, K. Formulation of the reaction coordinate. *J. Phys. Chem.* **1970**, *74*, 4161–4163.
5. Tomasi, J.; Persico, M. Molecular interactions in solution: and overview of methods based on continuous distributions of the solvent, *Chem. Rev.*, **1994**, *94*, 2027–2094.
6. Simkin, Y.B.; Sheikhet, I.I. *Quantum Chemical and Statistical Theory of Solutions–Computational Approach*, Ellis Horwood, London, **1995**.
7. Domingo, L.R. A new C–C bond formation model based on the quantum chemical topology of electron density. *RSC Adv.* **2014**, *4*, 32415–32428.
8. Reed, A. E.; Weinstock, R. B.; Weinhold, F. Natural population analysis, *J. Chem. Phys.* **1985**, *83*, 735–746.
9. Reed, E.; Curtiss, L.A.;Weinhold, F. Intermolecular interactions from a natural bond orbital, donor-acceptor viewpoint. *Chem. Rev.* **1988**, *88*, 899–926.
10. Parr, R.G.; Yang, W. *Density functional theory of atoms and molecules*, Oxford University Press, New York, **1989.**
11. Domingo, L.R.; Ríos-Gutiérrez, M.; Pérez, P. Applications of the conceptual density functional indices to organic chemistry reactivity. *Molecules,* **2016**, *21*, 748.
12. Becke A.D.; Edgecombe K.E. A simple measure of electron localization in atomic and molecular-systems, *J. Chem. Phys*. **1990**, *92*, 5397–5403.
13. Noury, S.; Krokidis, X.; Fuster, F.; Silvi, B. Computational tools for the electron localization function topological analysis, *Comput. Chem*. **1999**, *23*, 597–604.
14. Dennington, R.; Keith, T. A.; Millam, J. M., Semichem, Inc., Shawnee Mission, KS, *GaussView,* Version 6.1, Semichem Inc., Shawnee Mission, KS, **2016**.
15. Domingo, L. R.; Pérez, P.; Sáez, J. A. Understanding the local reactivity in polar organic reactions through electrophilic and nucleophilic Parr functions. *RSC Adv*. **2013**, *3*, 1486–1494.
16. Frisch, M. J.; Trucks, G. W.; Schlegel, H. B.; Scuseria, G. E.; Robb, M. A.; Cheeseman, J. R.; Montgomery, J. A.; Vreven, T. J.; Kudin, K. N.; Burant, J. C.; Millam, J. M.; Iyengar, S. S.; Tomasi, J.; Barone, V.; Mennucci, B.; Cossi, M.; Scalmani, G.; Rega, N.; Petersson, G. A.; Nakatsuji, H.; Hada, M.; Ehara, M.; Toyota, K.; Fukuda, R.; Hasegawa, J.; Ishida, M.; Nakajima, Y.; Honda, O.; Kitao, O.; Nakai, H.; Klene, M.; Li, X.; Knox, J. E.; Hratchian, H. P.; Cross, J. B.; Adamo, C.; Jaramillo, J.; Gom-perts, R.; Stratmann, R. E.; Yazyev, O.; Austin, A. J.; Cammi, R.; Pomelli, C.; Ochterski, J. W.; Ayala, P. Y.; Morokuma, K.; Voth, G. A.; Salvador, P.; Dannenberg, J. J.; Zakrzewski, V. G.; Dapprich, S.; Daniels, A. D.; Strain, M. C.; Farkas, M. C.; Malick, D. K.; Rabuck, A. D.; Raghavachari, K.; Foresman, J. B.; Ortiz, J. V.; Cui, Q.; Baboul, A. G.; Clifford, S.; Cioslowski, J.; Stefanov, B. B.; Liu, G.; Liashenko, A.; Piskorz, P.; Komaromi, I.; Martin, R. L.; Fox, D. J.; Keith, T.; Al-Laham, M. A.; Peng, C. Y.; Nanayakkara, A.; Challacombe, M.; Gill, P. M. W.; Johnson, B.; Chen, W.; Wong, M. W.; Gonzalez, C.; Pople, J. A. Gaussian 16 rev A.1 Gaussian Inc, Wallingford CT, 2016.

Table S1. ωB97X-D/6-311G(d,p) enthalpies, H in a.u., entropies S in cal·mol^{-1}·K^{-1} and Gibbs free energies, G in a.u., computed in benzene at 25 ºC, relatives to **13** + **14** or **15** + **14**, of the stationary points associated with the domino [4+2]/[3+2] cycloaddition reactions between DMF **13** and NS **14**.

	H	ΔH	S	ΔS	G	ΔG
**13**	-308.508199		81.78		-308.547053	
**14**	-438.745922		90.66		-438.788996	
**TS-1mn**	-747.250662	2.17	122.07	-50.37	-747.308659	17.19
**TS-1mx**	-747.239903	8.92	125.35	-47.08	-747.299461	22.96
**TS-1on**	-747.239369	9.26	125.77	-46.66	-747.299127	23.17
**15**	-747.314212	-37.71	119.46	-52.98	-747.370970	-21.91
**17**	-747.312596	-36.69	120.37	-52.07	-747.369785	-21.17
**18**	-747.319866	-41.26	119.40	-53.04	-747.376595	-25.44
**TS-21**	-1186.046626	8.48	168.26	-41.85	-1186.126573	20.95
**ZW**	-1186.059078	0.66	160.34	-49.77	-1186.135261	15.50
**TS-22**	-1186.058433	1.07	158.09	-52.02	-1186.133546	16.58
**16**	-1186.124413	-40.34	149.25	-60.86	-1186.195327	-22.19

Table S2. ωB97X-D/6-311G(d,p) computed total energies in benzene, single imaginary frequency of the TSs, and Cartesian coordinates of the stationary points involved in the domino [4+2]/[3+2] cycloaddition reactions between DMF **13** and NS **14**.

13

E(RwB97XD) = -308.642104300 A.U.

C	1.10129600	-0.00396600	-0.00004400
C	0.71735500	1.29444500	-0.00000900
C	-0.71735500	1.29444500	-0.00037700
C	-1.10129600	-0.00396600	0.00002600
O	0.00000000	-0.80572800	0.00022600
H	1.37456300	2.14997100	-0.00002100
H	-1.37456300	2.14997100	-0.00066300
C	-2.42141800	-0.68306500	0.00015900
H	-2.53956600	-1.31646800	-0.88339300
H	-2.53964000	-1.31600400	0.88403500
H	-3.21868700	0.06093400	-0.00007300
C	2.42141800	-0.68306500	-0.00002800
H	3.21868700	0.06093400	-0.00017400
H	2.53964800	-1.31610500	0.88377300
H	2.53955800	-1.31636700	-0.88365500

14

E(RwB97XD) = -438.886573269 A.U.

C	-1.95746600	1.41074800	0.57031300
C	-1.32864200	0.31664400	0.14072800
H	-1.41397000	2.27723700	0.92503500
O	-3.24818000	-0.68403700	-0.48107800
N	-2.09993600	-0.87686200	-0.19151300
H	-3.04061300	1.43316200	0.58943900
C	0.13323900	0.12231300	0.05907800
C	0.70229700	-1.13094800	0.30139900
C	0.96849900	1.19393800	-0.26661900
C	2.07935700	-1.29861400	0.23971600
H	0.06300700	-1.97049200	0.54278000
C	2.34413400	1.02293400	-0.32555000
H	0.53642700	2.16234100	-0.49394600
C	2.90409200	-0.22402500	-0.07044400
H	2.50898900	-2.27412900	0.43642900
H	2.97959900	1.86275000	-0.58201400
H	3.97848800	-0.35848500	-0.12023400

TS-1mn

E(RwB97XD) = -747.526679620 A.U.
Imaginary frequency -417.5066 cm^{-1}

C	1.03626600	0.52832700	-1.50706400
C	0.03918800	1.06026400	-0.69716800

H	1.83405100	1.20564400	-1.78425400
O	1.46911100	2.63921200	-0.06199700
C	2.71743700	0.18803500	0.56836200
C	2.21900100	-0.72789500	-0.37167000
N	0.31803900	2.20157600	0.03829000
H	0.78578200	-0.23542300	-2.23214800
C	0.92987800	-0.83198700	1.40475200
C	1.86725200	0.14116700	1.67059300
O	1.20230100	-1.43954200	0.24302800
H	1.87830500	0.77799100	2.54009800
H	3.55774800	0.84432000	0.41195000
C	2.98222700	-1.48144700	-1.41510500
H	3.60296800	-0.78953900	-1.98656900
H	2.30515300	-1.99205500	-2.10170900
H	3.63166000	-2.22802400	-0.95207300
C	-0.24162100	-1.31497800	2.16791900
H	-1.16242700	-1.10863900	1.61370600
H	-0.29053500	-0.81269000	3.13331300
H	-0.17636600	-2.39413600	2.32598000
C	-1.31164600	0.48884000	-0.53049500
C	-2.28024500	1.15538600	0.23295100
C	-1.66097400	-0.74307500	-1.09630700
C	-3.54318000	0.61120700	0.41596600
H	-2.02544200	2.10405300	0.68677500
C	-2.92625500	-1.28552100	-0.91128400
H	-0.93564900	-1.30402300	-1.67304600
C	-3.87711600	-0.61272500	-0.15452000
H	-4.27329400	1.14980600	1.01021500
H	-3.16639600	-2.24312500	-1.36012600
H	-4.86440300	-1.03614200	-0.00997500

TS-1mx

E(RwB97XD) = -747.515740852 A.U.
Imaginary frequency -450.9040 cm^{-1}

C	-0.42825400	-0.12593700	-0.92248200
C	0.67092200	0.68698700	-0.66312000
H	-0.27659500	-1.18698900	-1.07939700
O	-0.70957800	2.41480200	-0.60239100
C	-2.11501300	0.88350000	0.93294200
C	-1.52415600	-0.38217700	0.79309000
N	0.46716300	2.02675400	-0.46263400
H	-1.26919200	0.32582800	-1.43873000
C	-3.55344600	-0.48215800	-0.07494900
C	-3.38804800	0.81113400	0.33776000
O	-2.48570200	-1.22725200	0.25221900
H	-4.09577500	1.61288000	0.20297900
H	-1.67737700	1.72707000	1.43711800
C	-0.54494200	-1.03990400	1.71423000
H	0.25952800	-0.33914100	1.94238000
H	-0.10536700	-1.92710200	1.25586200
H	-1.03273300	-1.33170300	2.64734000
C	-4.65536900	-1.16530500	-0.79341900

H	-4.30919300	-1.54040200	-1.75984800
H	-5.47899600	-0.47221200	-0.96088100
H	-5.01953600	-2.01742100	-0.21415200
C	2.02926000	0.17663600	-0.38510300
C	2.47596100	-1.02999000	-0.93449600
C	2.90507500	0.89041700	0.44178500
C	3.74536400	-1.51691800	-0.65269700
H	1.83430000	-1.58822100	-1.60758700
C	4.17732500	0.40769100	0.71388200
H	2.57373200	1.82939600	0.86693400
C	4.60297200	-0.80116400	0.17416400
H	4.06966400	-2.45391100	-1.09179600
H	4.83833600	0.97657600	1.35846000
H	5.59539700	-1.17919000	0.39161500

TS-1on

E(RwB97XD) = -747.515263744 A.U.
Imaginary frequency -483.5533 cm^{-1}

C	-0.49002000	-1.77804400	0.72456300
C	0.22761800	-1.24132800	-0.34025500
N	-0.43450200	-0.98997900	-1.50464900
O	-1.64791100	-1.30525300	-1.49033400
C	-2.61440800	-0.02169500	0.25632700
C	-1.80547100	-0.28927000	1.37589600
H	0.00070200	-1.93692700	1.67703600
H	-1.31114400	-2.43992600	0.48002500
C	-1.21157300	1.66859800	0.45951700
C	-0.97019300	0.88760900	1.52376800
O	-2.24571500	1.14101900	-0.29149400
H	-0.20320400	1.04752200	2.26440200
H	-2.15038400	-0.91703800	2.18518100
C	-3.90820200	-0.61025100	-0.15880900
H	-3.91438500	-1.67700600	0.06477200
H	-4.05979100	-0.47315600	-1.22791400
H	-4.72840900	-0.13192200	0.38648400
C	-0.60869000	2.91965700	-0.05427400
H	-0.14290700	2.74790700	-1.02796400
H	0.15726300	3.26867200	0.63806500
H	-1.36486200	3.69993200	-0.17223900
C	1.58702400	-0.68074700	-0.21363000
C	1.99602900	0.38309300	-1.02678900
C	2.49340900	-1.18112500	0.72613000
C	3.26532500	0.92805200	-0.89840400
H	1.30234400	0.77407000	-1.76037200
C	3.75997300	-0.62721400	0.86193400
H	2.21734500	-2.02712600	1.34625800
C	4.15215000	0.43072800	0.05089400
H	3.56141700	1.75202600	-1.53833300
H	4.44696600	-1.03282500	1.59636300
H	5.14173400	0.86115100	0.15418700

15

E(RwB97XD) = -747.594010827 A.U.

C	-0.47738900	-1.49929300	-0.38444600
C	0.38986600	-0.72001400	0.56434900
H	-0.61941700	-2.50788300	0.01744900
O	-1.36313500	-0.80356000	1.95468100
C	-2.30946500	-0.42524100	0.93496200
C	-1.83891800	-0.81726800	-0.48560000
N	-0.05082800	-0.37732500	1.71587600
H	-0.03654600	-1.57390200	-1.37766900
C	-2.08066900	1.44234100	-0.39499800
C	-2.50836600	1.04942000	0.80493400
O	-1.63965300	0.43191200	-1.19214400
H	-2.88644000	1.69147300	1.58488000
H	-3.20103100	-0.97850300	1.23595900
C	-2.86296900	-1.62843100	-1.25503400
H	-3.04105500	-2.58438700	-0.75627900
H	-2.51302000	-1.81923500	-2.27184400
H	-3.80759900	-1.08180200	-1.30827900
C	-1.99827100	2.79394000	-1.00363700
H	-0.96101000	3.01826100	-1.26559200
H	-2.36280300	3.55062000	-0.30967400
H	-2.58899000	2.83302800	-1.92232900
C	1.76720400	-0.30726400	0.21528500
C	2.52953200	-1.04977000	-0.68791000
C	2.32977300	0.82897200	0.80393200
C	3.83091400	-0.66967000	-0.99129300
H	2.11801900	-1.94142000	-1.14713100
C	3.62731800	1.20861000	0.49640600
H	1.73334400	1.40854900	1.49737300
C	4.38219900	0.46086400	-0.40205000
H	4.41422900	-1.25965900	-1.68881600
H	4.05043700	2.09513200	0.95481400
H	5.39542000	0.76101900	-0.64368900

17

E(RwB97XD) = -747.592365857 A.U.

C	-0.31434700	-0.74062400	-0.52913000
C	0.71108300	0.35182800	-0.41116900
H	0.10484700	-1.73059900	-0.35667100
O	-1.03957100	1.71948700	-0.63663400
N	0.34601300	1.57880800	-0.47324500
H	-0.73153800	-0.70315000	-1.53983300
C	-3.62271300	-0.19319300	-0.15560500
C	-3.22781800	1.05770700	0.08801900
C	-1.77460000	1.05138400	0.40594300
C	-1.45502700	-0.46015100	0.45200400
O	-2.64757800	-1.13124500	-0.01827600
H	-3.84680900	1.93912100	0.03557800
H	-1.51014100	1.54252900	1.34929100

C	-1.16375700	-0.95592600	1.85645000
H	-0.23347900	-0.51730500	2.22765200
H	-1.06684300	-2.04344600	1.86045400
H	-1.97438500	-0.67362000	2.53272600
C	-4.95208700	-0.72853500	-0.54386700
H	-4.87712200	-1.25526300	-1.49863600
H	-5.67832700	0.07812200	-0.63804500
H	-5.30381900	-1.44444100	0.20328600
C	2.15433400	0.07674700	-0.22838200
C	2.98228100	1.03633600	0.36276600
C	2.71531800	-1.12766300	-0.65687900
C	4.33808300	0.79386600	0.51892700
H	2.54663000	1.96850200	0.69981400
C	4.07535700	-1.36646000	-0.50307000
H	2.09834100	-1.88087300	-1.13291300
C	4.88939000	-0.40862900	0.08725700
H	4.96739500	1.54386300	0.98417500
H	4.49807700	-2.30321800	-0.84741600
H	5.94930300	-0.59793200	0.21294200

18

E(RwB97XD) = -747.599537886 A.U.

C	-0.26855400	-1.39997100	0.97935300
C	0.44991200	-0.82498100	-0.20645600
H	0.30855500	-1.28668000	1.89626100
O	-1.47840700	-1.16816900	-1.30195900
N	-0.15163300	-0.68994100	-1.32682000
H	-0.43252600	-2.46880400	0.80456300
C	1.85037100	-0.35200900	-0.13265300
C	2.74724600	-0.92291700	0.77165300
C	2.29583400	0.66538500	-0.98098000
C	4.06699100	-0.49207600	0.82160200
H	2.42476300	-1.72177900	1.42988300
C	3.61209100	1.09750600	-0.92520400
H	1.59566500	1.11242400	-1.67542900
C	4.50166500	0.52038400	-0.02427200
H	4.75573300	-0.94942700	1.52250500
H	3.94496600	1.89187300	-1.58337000
H	5.52968500	0.86151300	0.01954500
C	-2.01291800	1.49957100	0.48493700
C	-1.52176000	0.76322600	1.47816500
C	-1.62430000	-0.68921000	1.11043200
C	-2.29502600	-0.62474500	-0.28729900
O	-2.49778300	0.77043800	-0.56352800
H	-1.08265400	1.14671400	2.38683500
H	-2.25509800	-1.25141300	1.80490100
C	-3.61759700	-1.34438900	-0.39845500
H	-3.48167800	-2.40867700	-0.19717700
H	-4.02424500	-1.21614600	-1.40211400
H	-4.31650600	-0.92832200	0.32934700
C	-2.12306700	2.97044500	0.32046400
H	-1.56776100	3.28910600	-0.56547300

H	-1.72558000	3.48448000	1.19534600
H	-3.16770400	3.25926200	0.17828900

TS-21

E(RwB97XD) = -1186.46928872 A.U.
Imaginary frequency -328.5079 cm^{-1}

C	-1.20913500	-0.22561100	0.48993800
N	-0.19612000	0.00991100	-0.26344200
C	0.85186200	-1.20106000	-1.16087600
C	1.94786600	-1.52231900	-0.33576200
N	1.64626900	-2.56603700	0.49187500
C	-1.42302600	0.66964100	1.67196100
H	-0.60397900	0.50557200	2.38033900
O	0.58673600	1.08304500	0.09342400
C	-0.17231100	2.31314400	0.25015600
C	-1.38279800	2.12347300	1.19606100
H	-2.37385400	0.46313800	2.16078100
C	-2.14154700	2.74209100	-0.85722100
C	-0.81555600	2.79098500	-1.00459800
O	-2.55412600	2.32818600	0.36952100
H	-0.28451700	3.09764600	-1.89174900
H	0.58585100	2.97502000	0.66854600
C	-1.42247800	3.12769600	2.32993500
H	-0.55518800	2.99729500	2.98131500
H	-2.33221600	2.99978200	2.91951100
H	-1.40756600	4.14295900	1.92718200
C	-3.23665300	3.06058700	-1.80641900
H	-3.86708100	2.17948500	-1.95145200
H	-2.83305000	3.37570700	-2.76784800
H	-3.86691900	3.85605000	-1.40167500
H	0.99423500	-0.58423700	-2.04000200
O	2.48874200	-2.93703400	1.32372100
H	0.10535700	-1.98123300	-1.25659800
C	-2.13054800	-1.31144300	0.13032200
C	-2.65429800	-2.14105200	1.12278500
C	-2.49183400	-1.51272900	-1.20373300
C	-3.51089000	-3.17605400	0.77921000
H	-2.36903300	-1.99667800	2.15852100
C	-3.35743000	-2.54297000	-1.54069900
H	-2.10537800	-0.84628600	-1.96643200
C	-3.86385500	-3.37728100	-0.55073500
H	-3.90038400	-3.82989800	1.55011400
H	-3.63993900	-2.69236600	-2.57597600
H	-4.53742800	-4.18397700	-0.81518900
C	3.18815800	-0.71719300	-0.31639200
C	3.66987400	-0.13210400	-1.49496300
C	3.90976900	-0.49216300	0.86301000
C	4.81079200	0.65732800	-1.49775000
H	3.15267500	-0.30822500	-2.43255800
C	5.05369200	0.29637600	0.85910100
H	3.57209600	-0.95325900	1.77921400
C	5.51112500	0.87810400	-0.31691100

H	5.15997600	1.09334200	-2.42773800
H	5.59308500	0.45530100	1.78699100
H	6.40671800	1.48950100	-0.31618100

ZW

E(RwB97XD) = -1186.483844040 A.U.

C	-1.89236100	-0.44609300	-1.72264400
C	-0.78372700	-0.09441300	-0.77805600
N	1.52126300	1.00761900	1.91760000
N	-0.51892300	-0.97823100	0.12143700
C	0.32439600	-0.91188800	1.39660500
H	-1.55632200	-1.27456900	-2.35462000
O	-1.22975600	-2.15070700	0.04531500
C	-2.68188400	-1.92557200	0.13290700
C	-3.12283400	-0.89316000	-0.92649700
H	-2.12726200	0.40707600	-2.35609300
C	-3.56571600	-0.06887500	1.14892300
C	-3.10870100	-1.29851000	1.40661100
O	-3.58217400	0.25347400	-0.17221300
H	-3.02331500	-1.75242900	2.38086300
H	-3.05314000	-2.93082100	-0.06223400
C	-4.25986200	-1.36965000	-1.80658100
H	-3.94169700	-2.22589100	-2.40558700
H	-4.58238100	-0.56903200	-2.47433500
H	-5.10756600	-1.67011200	-1.18718200
C	-4.04487800	0.99829700	2.05902400
H	-3.38783100	1.86862700	1.98232900
H	-4.04735700	0.64933600	3.09050700
H	-5.05270300	1.31109600	1.77726400
C	1.60380200	-0.18551400	1.31634200
H	0.39955800	-1.96916500	1.65411900
O	2.51815900	1.77855400	1.94280800
H	-0.32703600	-0.40263000	2.10929400
C	-0.13521900	1.22676400	-0.85587700
C	1.20462200	1.36683900	-1.20621900
C	-0.92895200	2.34794100	-0.61563200
C	1.76260100	2.63353300	-1.26277200
H	1.81343900	0.49445000	-1.40377600
C	-0.35690200	3.61111500	-0.66649600
H	-1.97780600	2.22547900	-0.36884200
C	0.98757200	3.75332200	-0.98457800
H	2.81265500	2.74007700	-1.50325200
H	-0.96228500	4.48326600	-0.45066200
H	1.43529500	4.73959300	-1.01069900
C	2.73430800	-0.78862700	0.61273400
C	2.58141700	-2.01632300	-0.06494100
C	4.00001300	-0.17263100	0.53499600
C	3.62658100	-2.59664900	-0.76936600
H	1.63045600	-2.53851900	-0.04933800
C	5.03929400	-0.76090400	-0.17357300
H	4.12866800	0.77216900	1.04103300
C	4.86757000	-1.97317300	-0.83211100

H	3.46767300	-3.54443400	-1.27363300
H	6.00146300	-0.25975800	-0.20883200
H	5.68444200	-2.42507300	-1.38366500

TS-22

E(RwB97XD) = -1186.48253738 A.U.
Imaginary frequency -49.1297 cm^{-1}

C	-2.12079900	-0.20070300	-1.56852100
C	-1.08405000	0.32725600	-0.63023600
N	1.59859300	0.15361300	-1.56811300
N	-0.36278700	-0.57047700	-0.04525200
C	0.89058200	-0.34886100	0.65367100
H	-1.58223100	-0.58236200	-2.44243200
O	-0.52209300	-1.87048100	-0.45244100
C	-1.89586900	-2.32854700	-0.26131400
C	-2.89543000	-1.34625300	-0.91336400
H	-2.79352300	0.59311600	-1.88710800
C	-3.29555800	-1.44091700	1.32742100
C	-2.33475200	-2.35285100	1.15776700
O	-3.64694000	-0.78180100	0.19038400
H	-1.92196000	-2.99091900	1.92278300
H	-1.86046800	-3.29313600	-0.76639300
C	-3.87175700	-2.01856500	-1.85655800
H	-3.33898500	-2.44412000	-2.70982400
H	-4.60559200	-1.29730000	-2.21995900
H	-4.39855400	-2.82108000	-1.33561700
C	-4.03805000	-1.02567700	2.54226500
H	-3.85881800	0.03414400	2.74216000
H	-3.71886900	-1.60868200	3.40503000
H	-5.11168400	-1.15985700	2.39181800
C	2.00863200	-0.20549900	-0.37178600
H	1.02609800	-1.21863500	1.29724600
O	2.40068700	0.37809300	-2.53189100
H	0.75457800	0.53506200	1.28753800
C	-1.05782000	1.75312100	-0.23995100
C	0.04332700	2.58784600	-0.42626500
C	-2.23657000	2.25752000	0.32117500
C	-0.03171300	3.91411400	-0.02033900
H	0.92588400	2.19973700	-0.91661400
C	-2.29292600	3.57900600	0.73619700
H	-3.09580100	1.60664300	0.44289800
C	-1.18928900	4.40858200	0.56679600
H	0.82217900	4.56298800	-0.17305500
H	-3.20137400	3.96249800	1.18530000
H	-1.23741300	5.44446000	0.88204300
C	3.37570300	-0.42005400	0.10119400
C	3.65091600	-0.58751900	1.47198300
C	4.46935700	-0.45183500	-0.78509300
C	4.94439300	-0.78738400	1.93290300
H	2.85124100	-0.55039900	2.20393900
C	5.75944000	-0.65345500	-0.31449500
H	4.26684300	-0.30294100	-1.83547800

C	6.01091400	-0.82434000	1.04239400
H	5.11888000	-0.91094200	2.99673400
H	6.58151100	-0.67642300	-1.02261400
H	7.02208500	-0.98019900	1.40168000

16

E(RwB97XD) = -1186.551432430 A.U.

C	1.23245000	-0.51233000	1.55790500
C	0.38435100	0.11399200	0.42830800
N	0.41689100	-0.68235600	-0.80316400
C	-0.93895300	-0.56211700	-1.36893800
H	0.57681300	-1.13286100	2.17353100
O	0.52053300	-2.08261000	-0.41587300
C	1.88860900	-2.36351200	-0.05298100
C	2.38285500	-1.38112900	1.04924700
H	1.62576400	0.27771300	2.20140300
C	3.67544600	-1.11395200	-0.82051200
C	2.90771800	-2.16851300	-1.13208400
O	3.37602100	-0.53527100	0.38645100
H	2.97106300	-2.75465100	-2.03898800
H	1.81067500	-3.38878400	0.32423700
C	3.09935800	-2.09916700	2.19119400
H	2.40779500	-2.75622200	2.72958400
H	3.51653400	-1.37553200	2.89866700
H	3.91884700	-2.70844300	1.79535700
C	4.79587400	-0.46344000	-1.55813700
H	4.56836800	0.59419700	-1.73676100
H	4.96693700	-0.95617700	-2.51826800
H	5.72039400	-0.50011200	-0.96969400
N	-1.10036500	0.05533100	0.83588600
C	-1.84839600	-0.29078400	-0.19139200
H	-1.17273200	-1.48740500	-1.89834600
O	-1.43439500	0.34649700	2.03331100
H	-0.94800900	0.26287500	-2.09319200
C	0.74267400	1.57250500	0.12437100
C	0.39490600	2.58517200	1.03101000
C	1.44456500	1.91176900	-1.03901000
C	0.73987400	3.91121600	0.77173000
H	-0.16250300	2.32977400	1.92602500
C	1.78726600	3.24155500	-1.29440600
H	1.72076300	1.12953000	-1.73651200
C	1.43791800	4.24537900	-0.39108500
H	0.45962000	4.68523000	1.48128900
H	2.33011600	3.48967800	-2.20286200
H	1.70666100	5.27941500	-0.59011000
C	-3.29997100	-0.36212900	-0.21279400
C	-3.94789300	-0.70043100	-1.42085300
C	-4.09477400	-0.10219600	0.92527500
C	-5.33559600	-0.77569500	-1.49026200
H	-3.36369000	-0.90197300	-2.31365600
C	-5.48294700	-0.18263700	0.84462500
H	-3.60533900	0.15846500	1.85402500

C	-6.11114600	-0.51831100	-0.35692300
H	-5.81235900	-1.03633000	-2.43127400
H	-6.07910500	0.01930100	1.73064000
H	-7.19469000	-0.57891100	-0.41097700
